# One man’s trash is another man’s treasure. Interdisciplinary examination of taphonomic aspects of ceramic sherds, animal bones and sediments from the La Tène period settlement at Basel-Gasfabrik

**DOI:** 10.1371/journal.pone.0236272

**Published:** 2020-07-27

**Authors:** David Brönnimann, Johannes Wimmer, Milena Müller-Kissing, Barbara Stopp, Hannele Rissanen, Norbert Spichtig

**Affiliations:** 1 Integrative Prehistory and Archaeological Science, University of Basel, Basel, Switzerland; 2 Archaeological Service of Canton Basel-Stadt, Basel, Switzerland; 3 Department of Prehistoric Archaeology, Institute of Archaeological Sciences, University of Bern, Bern, Switzerland; 4 Institute of Archaeological Studies, Faculty of Historical Sciences, Ruhr-University of Bochum, Bochum, Germany; University at Buffalo - The State University of New York, UNITED STATES

## Abstract

As part of an interdisciplinary research project on the Late La Tène period settlement at Basel-Gasfabrik, ceramic sherds, animal bones and archaeological sediments from different archaeological structures (one large pit, two ditches and four archaeological layers) were examined in respect of 21 taphonomic features (proxies). These proxies, in turn, were linked to different processes that can leave traces on objects or sediments: primary use, mechanical stress, heat impact, water, redeposition, exposure, covering and postdepositional processes. The different proxies were compared using a statistical procedure. Our results show significant differences between the different features with regard to taphonomic alteration. For example, ceramic sherds and animal bones from archaeological layers show severe alteration due to exposure, whilst a good and uniform preservation within the pit points to its rapid filling. Furthermore, there is evidence of middens which probably served as material depots. Our results suggest that waste was not simply seen as rubbish, but was stored as a resource. Therefore, materials could take different “paths”, each of which resulted in specific taphonomic processes (alterations). The interdisciplinary approach taken in this project has provided new insight into the complex but probably clearly defined handling of various materials at Basel-Gasfabrik, thus allowing us to visualise part of the *cultural biography of things*.

## 1. Introduction

The interpretation of finds associations requires insight into site formation processes [1–3] as well as patterns of social behaviour such as waste disposal practices [4, 5] or the exploitation of resources. Archaeological objects and sediments, on the other hand, provide a wide range of information. This includes aspects of taphonomic alteration, which manifest themselves as changes on the artefact and allow us to draw conclusions concerning the way in which the object was treated and what kinds of depositional, redepositional and postdepositional processes were involved. The reconstruction of taphonomic processes can therefore be considered a promising approach with a high potential to provide new insight. A number of taphonomic analyses of different object groups and materials have been carried out in the context of (prehistoric) settlements. However, in most cases only single aspects of an object group have been investigated, such as the degree of fragmentation of pottery [6–9], whilst the examination of different taphonomic features [10, 11] or the integration of several disciplines is rare [e.g. 12].

As part of an interdisciplinary research project on the Late La Tène period settlement at Basel-Gasfabrik, ceramic sherds, animal bone fragments and sediments from one pit, two ditches and four archaeological layers (Table 1) were examined in respect of 21 taphonomic criteria (proxies, Tables 2–4). Each proxy was linked to one or more previously defined processes (Tables 5 and 6) that can result in the phenomenon observed and compared with each other using statistical methods. The processes evaluated included: primary use, mechanical stress, heat impact, water, redeposition, exposure, covering and postdepositional processes (Table 5). “Mechanical stress”, “redeposition” and “exposure” were grouped together under "physical alteration”.

**Table 1:** List of features, archaeological horizons and stratigraphic units (SU) examined as part of the taphonomic investigation and the underlying data.

Feature	Stratigraphic unit (SU)^a^	Description	No. of sherds	No. of bones	Microm. samples
**Pit 287**	2089	Bottom intentional fill	693	1134	1
	2089/2090	Mixed stratigraphic unit	30	140	
	2090	Middle intentional fill	259	655	3
	2090/2091	Mixed stratigraphic unit	224	720	
	2091	Top intentional fill	658	1561	
	2091/2092	Mixed stratigraphic unit	164	412	
	2092	Subsided layer of pebbles	23	62	
	2092/2093	Mixed stratigraphic unit	140	470	
	2093	Subsided layers	158	522	
**Ditch 7**	2007	Bottommost natural fill	99	159	2
	2007/2008	Mixed stratigraphic unit	42	71	
	2008	Bottom intentional fill	107	178	
	2008/2009	Mixed stratigraphic unit	109	121	
	2009	Top intentional fill	147	289	
**Ditch 9**	2031	Bottommost natural fill	175	307	1
	2031/2032	Mixed stratigraphic unit	159	278	
	2032	Bottom intentional fill	271	548	
	2033	Layer of debris			
**Archaeological horizons**	2015	aH1 Areas 1 and 2	336	1242	11
	2003/2004/2005 2086/2087/2088	aH2 Area 1	757	1616	5
	2002	aH3 Area 1	473	995	3
	2001	aH4 Area 1	142	120	2

^a^ Mixed stratigraphic units (e.g. SU 2089/2090 etc.) were defined to incorporate finds that could not be definitively associated with a specific stratigraphic unit.

**Table 2:** List of taphonomic proxies recorded during study of the pottery.

Proxy	Data type	Commentary
Fragmentation (fragment weight)	metric	Logarithmic fragment weight of fine ware wall sherds.
Surface preservation	categorical	Classification of the state of preservation of the outer surfaces of fine-ware fragments into five categories.
Painting (preserved on surface)	yes/no (categorical)	Presence of painting on oxidised fine wares.
Matching fragments	yes/no (categorical)	Are there conjoining sherds and can the fragments be associated with a distinguishable individual vessel?
Traces of burning	categorical	Classification of traces of burning on fine ware into four categories.
Spalling	yes/no (categorical)	Presence of surface spalling.
Rim, bottom and wall sherds	yes/no (categorical)	Does the fragment belong to the rim, bottom or wall of a vessel?

**Table 3:** List of taphonomic proxies recorded during study of the animal bones.

Proxy	Data type	Commentary
Severe traces of burning	categorical	Classification of the traces of burning into pre-defined categories.
Gnawing marks (carnivores)	yes/no (categorical)	Presence of carnivore gnawing.
Surface preservation	categorical	Classification of the state of surface preservation into three categories.
Fragmentation (average cattle bone weight)	metric	Number and fragment weight of cattle bones.
Rounded breaks	yes/no (categorical)	Presence of rounded breaks. Subjective proxy.
Root damage	categorical	Classification of the intensity of root damage into four categories.

**Table 4:** List of taphonomic proxies recorded in sediments and on bone fragments from block samples (histotaphonomy).

Proxy	Data type	Commentary
Fine lamination	categorical	Fine lamination of a layer; classification into six categories (from none to very clear lamination).
Horizontal alignment	categorical	Horizontal alignment of (micro-) components (e.g. microcharcoal); classification into six categories.
Compaction	categorical	Assessment of the compaction of a layer; classification into six categories.
Homogenisation	categorical	Homogenisation (e.g. due to bioturbation, trampling etc.) of a layer; classification into six categories (from none to very clear homogenisation).
Charcoal fragmentation	categorical	Ratio between microcharcoal (< 1 mm) and charcoal pieces (> 1 mm); classification into five categories.
Faeces fragmentation	categorical	Ratio between flecks of faeces (< 1 mm) and faeces remains (fragments of coprolites; > 1 mm); classification into five categories.
Fungal attack on bone	categorical	Intensity of fungal attack (Wedl Tunnel Index; classification into six categories) after [29].
Bone collagen content	categorical	Preservation of the collagen content (collagen index; classification into five categories) after [29]).

**Table 5:** Overview of eight pre-defined taphonomic processes.

Code ^a^	Process	Description and examples
**1**	**Primary use**	Use of the material/object in its original function.
**2**	**Mechanical stress**	Various causes of mechanical alteration to an object/sediment (e.g. trampling, reuse, recycling, knocks etc.).
**3**	**Heat impact**	Coincidental or intentional alteration of an object by heat/fire.
**4**	**Water**	Alteration by stagnant or flowing water. In this study, this particular process was only detected in sediments.
**5**	**Redeposition**	Intentional (e.g. by recycling, reuse, levelling, filling, cleansing etc.), coincidental (e.g. by trampling) or natural (e.g. by erosion or water) redeposition.
**6**	**Exposure**	Various causes of alteration (e.g. the elements, gnawing, bioerosion etc.) to an object/sediment due to exposure.
**7**	**Covering**	Rapid covering of an object/sediment, which protects it from various processes (mechanical stress, exposure, redeposition).
**8**	**Postdepositional processes**	Collective term for a multitude of different postdepositional changes (e.g. bioturbation, precipitation, geochemical processes etc.), which in the widest sense also include the archaeological examination.

^a^ The same colour code has been used in Figs 7–10.

**Table 6:** Linkage and weighting of proxies and processes.

Proxies	1) Primary use	2) Mechanical stress	3) Heat impact	4) Water	5) Redeposition	6) Exposure	7) Covering	8) Postdepositional processes
**Pottery**								
Fragmentation (high fragment weight)		-3					3	-1
Poor surface preservation		2	2			2	-3	
Painting (preserved on surface)		-2	-1			-2	3	
Matching fragments		-2	-1		-3		2	
Traces of burning			3				-1	
Spalling	2					2		1
Rim and bottom sherds		-2						
**Animal bones**								
Rounded breaks		2						
Poor surface preservation						2	-2	2
Gnawing marks (carnivores)						2		
Severe traces of burning			3					
Root damage								2
Fragmentation (average cattle bone weight)	-2	-1						
**Sediments**								
Fine lamination		3		3				
Homogenisation		2				2		3
Horizontal alignment		2		2		2		
Compaction		3				1		
Low charcoal fragmentation	3	-3						
Low faeces fragmentation		-3			-2	-3		
Low fungal attack on bone						-3	2	
High bone collagen content			-3			-2	2	

Negative values represent counter-movements of proxies and processes. Increased mechanical stress, for instance, results in reduced average fragment weights (“fragmentation”).

This novel approach allowed us to explore our overall research question, namely the identification of similarities or differences with regard to the taphonomic alteration between and within various material categories (pottery, animal bones and sediments) and between different features (one large pit, two ditches and four archaeological layers). This, in turn, formed the basis upon which the differentiated and complex way of treating the individual materials could be reconstructed.

### 1.1 Taphonomy in archaeology and theoretical approaches

The term taphonomy stems from palaeontology and was adapted by archaeologists as part of the *New Archaeology* movement to describe the study of depositional processes [e.g. 4]. Michael Schiffer’s work [1–3] played an important role in this. Schiffer strove to understand the processes that lead to the formation of archaeological remains (site formation processes) by tracing the artefacts’ “life cycles” [1]. During the course of an object’s life cycle, it is “transformed” in various ways and these processes were divided by Schiffer into non-cultural (n-transforms) and cultural (c-transforms) transformations. This was based on the notion that archaeological features are not the result of individual acts but of regular (everyday) activities of groups of people, in other words, of superordinate principles of social organisation (behavioural archaeology) [13]. This includes, among other things, waste disposal practices and resource management, both of which have a considerable influence on the distribution of the archaeological objects and the possibilities for their interpretation [4, 5].

**Fig 1:**
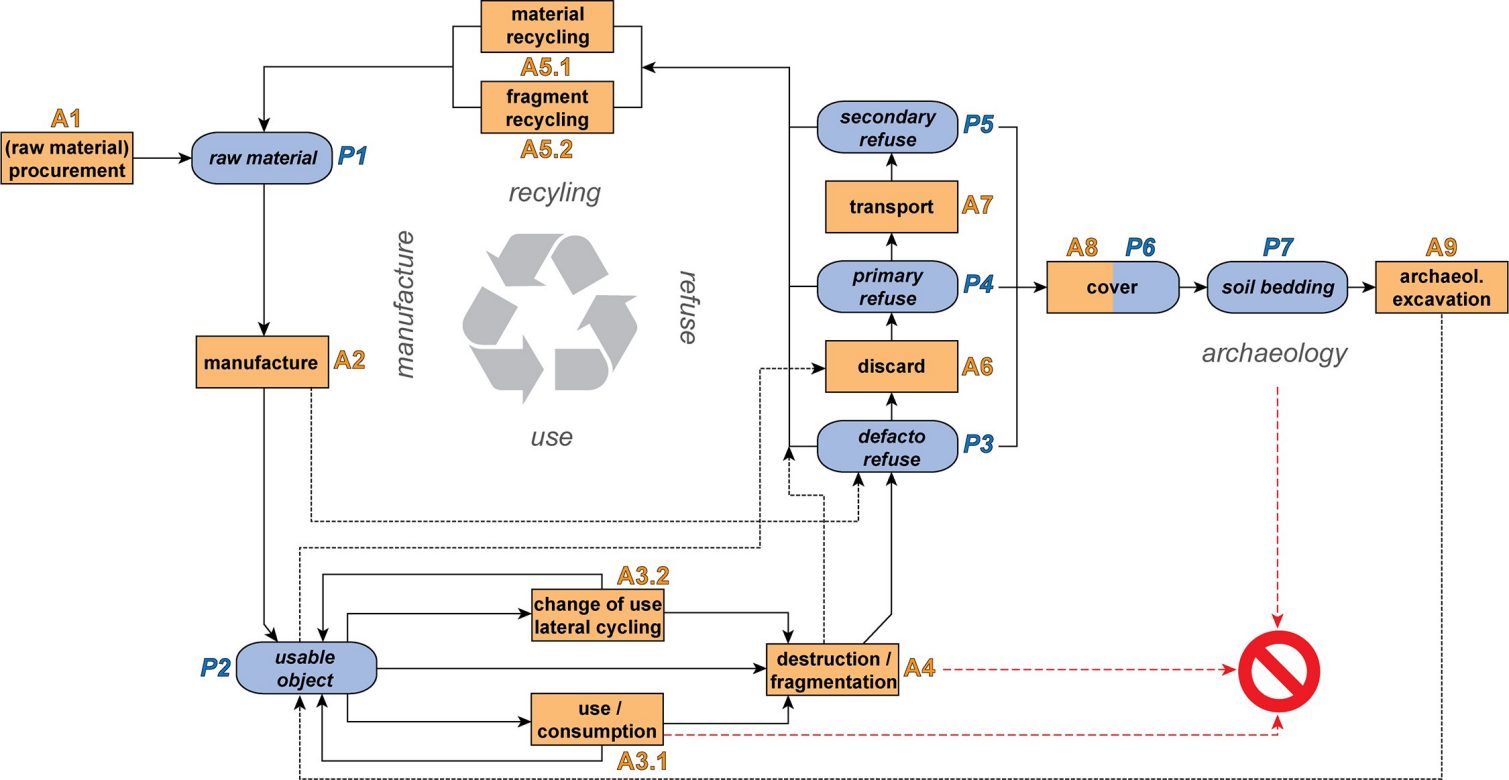
Flowchart showing the “life cycle” of objects or materials. Flow model based on Schiffer [1] and extended to reflect in a somewhat simplified manner the genesis, use, re-use and recycling as well as the disposal, destruction and deposition of an object in the ground and to illustrate this as part of a partially closed cycle. The “passive phases” are marked in blue, the “activated phases” in orange. The red symbol signifies the destruction or dissolution of the object.

In the 1980s, Igor Kopytoff coined the term “cultural biography of things”, which defined the treatment of a group of objects as a consequence of regular patterns of behaviour [14]. Despite the criticism and the charge of increased “popularisation” and oversimplification of the term “object biography” that has since been voiced [15–18], we have borrowed from Kopytoff’s idea. It is not our intention, however, to examine the lineally constructed and simplified “fates” of individual objects but to study the culturally determined treatment of certain object groups. Because a cultural biography of things does not necessarily progress along a straight line but sometimes moves in cycles and can involve several pathways [16], we have proposed an adapted version of Schiffer’s life cycle as a research tool (Fig 1). The flowchart retains the idea of “moving” and “static” phases [1, 16], though we have attempted to use words that are as neutral as possible and have therefore abstained from the use of palaeontological terms [see e.g. 19]. We have therefore proposed a distinction between “activated” and “passive” phases; in the activated phase the object is involved in an action, whereas in the passive phase it is not. It should be noted that an object can also undergo changes and transformations during a “passive” phase. Our flowchart allows us to reconstruct cyclical, linear and intertwined biographies. We have also incorporated the destruction or decay of objects (Fig 1). Our terminology for naming the different categories of waste follows Schiffer’s definitions. These include the terms de facto refuse (“Pompeii premise”; deposition of an object or material where it was used), primary refuse (an object or material “exits” the cycle) and secondary refuse (redeposited waste) [1].

The model of a (cyclic) flowchart presented here also illustrates that it is not possible, in an archaeological context, to assign objects or sediments to "living" or "dead" stages. For the purposes of this article, we therefore do not understand "taphonomic processes" as referring exclusively to post-sedimentary processes, but to all processes that can affect objects or sediments at any time.

### 1.2 Research goals

This paper deals with the question of which taphonomic features (proxies) can be identified on ceramic sherds, animal bones and sediments and which processes they can be linked with. Based on a quantification and evaluation of selected proxies, the project examined whether there were recognisable differences between the three material categories and between different archaeological features (pit 287, ditches 7 and 9), layers (aH1-aH4) and areas with regard to taphonomic alteration. Furthermore, the goal was to ascertain whether certain patterns could be detected which would allow us to reconstruct a cultural biography of things and material groups. This was based on the idea that most archaeological occupation deposits are ultimately a product of a general notion of everyday life, i.e. of systematic and often complex social practices. This includes, among other things, the handling of waste, which consequently leads to structured waste disposal [20–22]. This, in turn, is the basis for recognising structured deposits, i.e. "special" objects or object groups within a specific context or among large quantities of objects.

### 1.3 The Basel-Gasfabrik site

**Fig 2:**
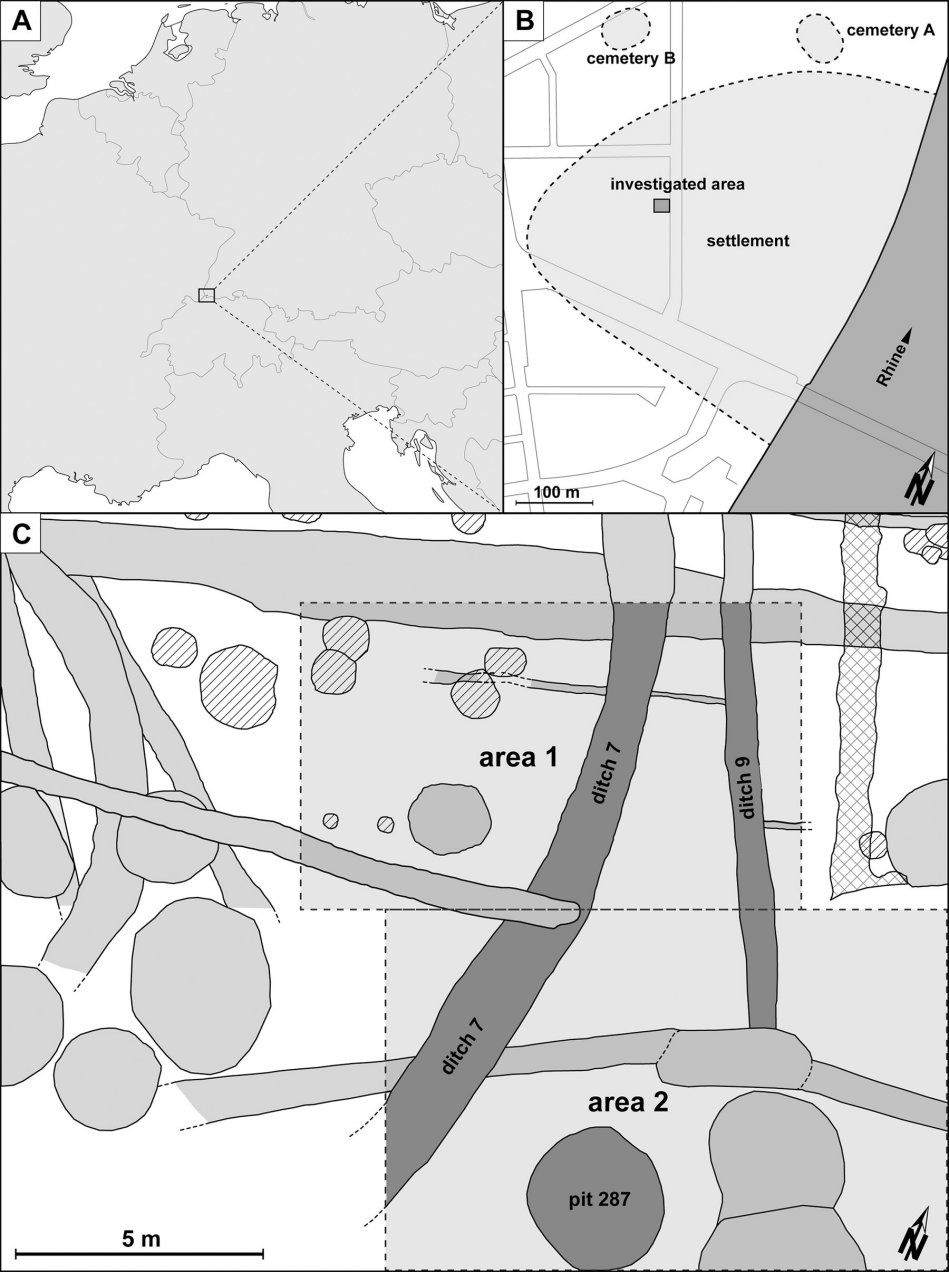
Location of the study area. The settlement of Basel-Gasfabrik lies in north-western Switzerland (A) on the left bank of the River Rhine in the city of Basel. The area examined (dark-grey zone) is situated in the western part of the settlement (B). Bottom (C): Plan of the examined areas and features. The ditch sections (ditch 7 and 9) and pit 287 are marked in dark-grey. The finds from the archaeological horizons aH1-aH4 were examined in area 1, whilst in area 2 only those from aH1 were analysed. Hatching and cross-hatching denotes later intrusions and disturbances.

The site of Basel-Gasfabrik (Switzerland, Fig 2A) was discovered in 1911 and is located 2 km north of today’s Basel city centre on the banks of the River Rhine (Fig 2B) in an area, which was protected from flooding. Stretching over c. 15 hectares, the unfortified central settlement dated from the Late La Tène period (200/150–80 BC) [23]. Countless coins and Mediterranean wine amphorae emphasised its importance within a long-distance trade network [24, 25]. Two associated cemeteries with more than 200 inhumations were situated north of the settlement [26]. Large parts of the settlement have been excavated, with the bulk of the work taking place between the 1980s and 2010s. The research tradition that has been developed over the course of this extended period has been very much focused on interdisciplinary cooperation. It has produced an extraordinary wealth of data and provided extensive archaeological, biological and geoarchaeological insight [26, 27, 9, 28, 29]. Included in this was an interdisciplinary investigation of two large pits [pits 283 and 321]. The infilling processes were reconstructed by geoarchaeological means and initial taphonomic investigations of ceramic sherds and animal bones were carried out. This resulted in the hypothesis that objects deposited in pits are generally better preserved than those found elsewhere within the settlement [30].

The features recorded over the years comprised almost 600 large pits, which had probably served mainly as silos and cellars and yielded a substantial number of finds. These objects, however, were most probably parts of secondary fills and therefore cannot be directly linked with the primary function of the pits. Geoarchaeological investigations have also shown that some pits had a secondary use as covered workshops (probably in the context of craftworking) [30]. Another category of features that is frequently found are ditches, which came to light in many areas of the settlement. Their function has not yet been fully understood. One important purpose, however, may have been to drain off surface water. Some ditches may also have served to divide the settlement into different areas [30]. Other features included a small number of wells and numerous post pits. Nevertheless, reconstructing house plans proved very difficult, although micromorphological investigations did identify rammed earth floors. The analyses revealed that no (micro-) remains (de facto refuse) could be detected directly above these house floors [30]. It is therefore assumed that the floors were kept clean or covered. Accordingly, no object inventories of individual houses were identified, nor have any places, streets or paths been located so far.

Remains of (stratified) archaeological layers only survived in natural depressions in the ground. The well-preserved archaeological layers in the area investigated were divided into four archaeological horizons (aH1-aH4) that differed from each other in their composition and layer formation processes [30]. All these features contained more than 700,000 artefacts and 1.6 million animal bones; several hundred geoarchaeological samples were also taken.

## 2. Features

A well-preserved section in the western area of the settlement was selected for the purposes of this study (Fig 2B). Located in an extended natural depression, it yielded a sequence of archaeological layers measuring a total of c. 50 cm in thickness [30, 31]. Four archaeological horizons (aH1-aH4) extending over the entire section were identified during the excavations, which took place in 1990 and 2002 respectively. Ditches, large pits and post pits were also recorded (Fig 2C) [32, 33]. The finds from pit 287, a 15.5 m long section of ditch 7 and a 10 m long section of ditch 9 were selected to record the taphonomic features of the animal bones and ceramic sherds (Fig 2C). Due to the large quantity of artefacts, examination of the finds from the archaeological horizons was restricted to an area of c. 60 m^2^ in size (Area 1). In respect of aH1, a second area measuring c. 88 m^2^ to the south-east was also added (Area 2; Fig 2C). Stratigraphic units (SU) representing the fills from pit 287 and from ditches 7 and 9 as well as the archaeological horizons were defined for the purposes of horizontal and vertical structuring (Table 1). With that, the most frequent types of structure in this area were selected for taphonomical analysis. Since there none of the objects or sediments were associated with specific houses or buildings (see chapter 1.3 in this paper), “house communities” or “craftworking units” could not be defined.

### 2.1 Stratigraphy and archaeological horizons

The Basel-Gasfabrik site is located on a low terrace of the River Rhine, which was formed in the last glacial period and consists of calcareous, sandy gravel [34, 35]. On top of this gravel, a silty-sandy loam (overbank deposit) measuring several tens of centimetres in thickness had accumulated, on which a luvisol with a characteristic Al-Bt-Cv-C soil profile had formed during the Holocene [31, 36]. Today, however, the Al horizon of this pre-La Tène period soil profile, and in some areas the Bt horizon, are missing. This can be attributed to truncation of the terrain before the archaeological horizons aH1-aH4 were deposited [30].

**Fig 3:**
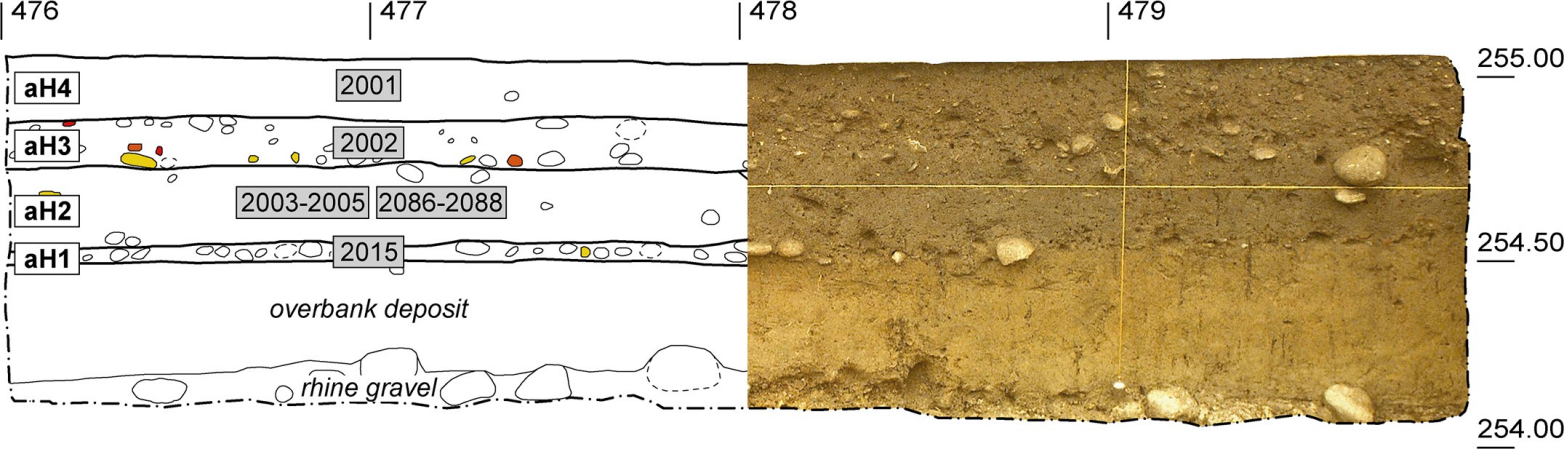
Archaeological horizons. Photo (right part) and drawing (left part) of an exemplary section. Apart from the natural Rhine gravel and the overbank deposit, the stratigraphy was divided into four archaeological horizons aH1 to aH4. Yellow objects: bones; red objects: ceramic sherds.

The stratigraphic sequence of the archaeological strata outside of pits and ditches bore close similarities throughout the whole area examined and was divided into four main deposits (archaeological horizons aH1-aH4) (Fig 3). Micromorphological examinations revealed that these differed from each other not just by virtue of their characteristics (texture, microfabric, composition and embedded microfinds) but also due to the sedimentation processes that had led to their formation [30].

Horizon **aH1** (SU 2015) can be described as a partially diffuse gravel horizon (Fig 3) measuring 5–10 cm in thickness throughout the area examined. It was interpreted as an initial trampling horizon, which had formed following the truncation of the natural soil profile by repeated trampling and reinforcement of the surface, which had at times probably been quite muddy. A layer of loam (**aH2**), which was dark in colour due to the presence of microcharcoal was found overlying aH1 (Fig 3). The micromorphological examination revealed localised differences both with regard to its composition and microstructure [30]. South-west of ditch 7, aH2 was 20–25 cm thick, had an homogenous fabric and contained both faeces and ash (SU 2003, 2004, 2005), which possibly points to the practice of horticulture. North-east of ditch 7, on the other hand, aH2 was only 15 cm thick (SU 2086, 2087, 2088). In contrast to the south-western area, there was no ash or faeces present, but rather fine lamination indicating gradual accumulation.

Horizon **aH3** (SU 2002) was up to 30 cm thick in the south-east and consisted mainly of pebbles from the Rhine, which had been selected for their size (Fig 3). Natural sedimentation could be excluded, as fluvial processes would have been the only possible options, and they would have had a completely different appearance (stratified, sandy-gravelly deposits in erosion channels). Horizon aH3 gradually became more diffuse towards the north and west, contained gravel and was only 15–20 cm thick. The stratigraphic sequence ended with **aH4** (SU 2001), which, based on its numerous modern finds and its strikingly homogenous appearance, was identified as a modern-era plough horizon (Fig 3). The La Tène period finds, which were also found in aH4, suggest that the area continued to be settled after aH3 had been deposited.

### 2.2 Pit 287

**Fig 4:**
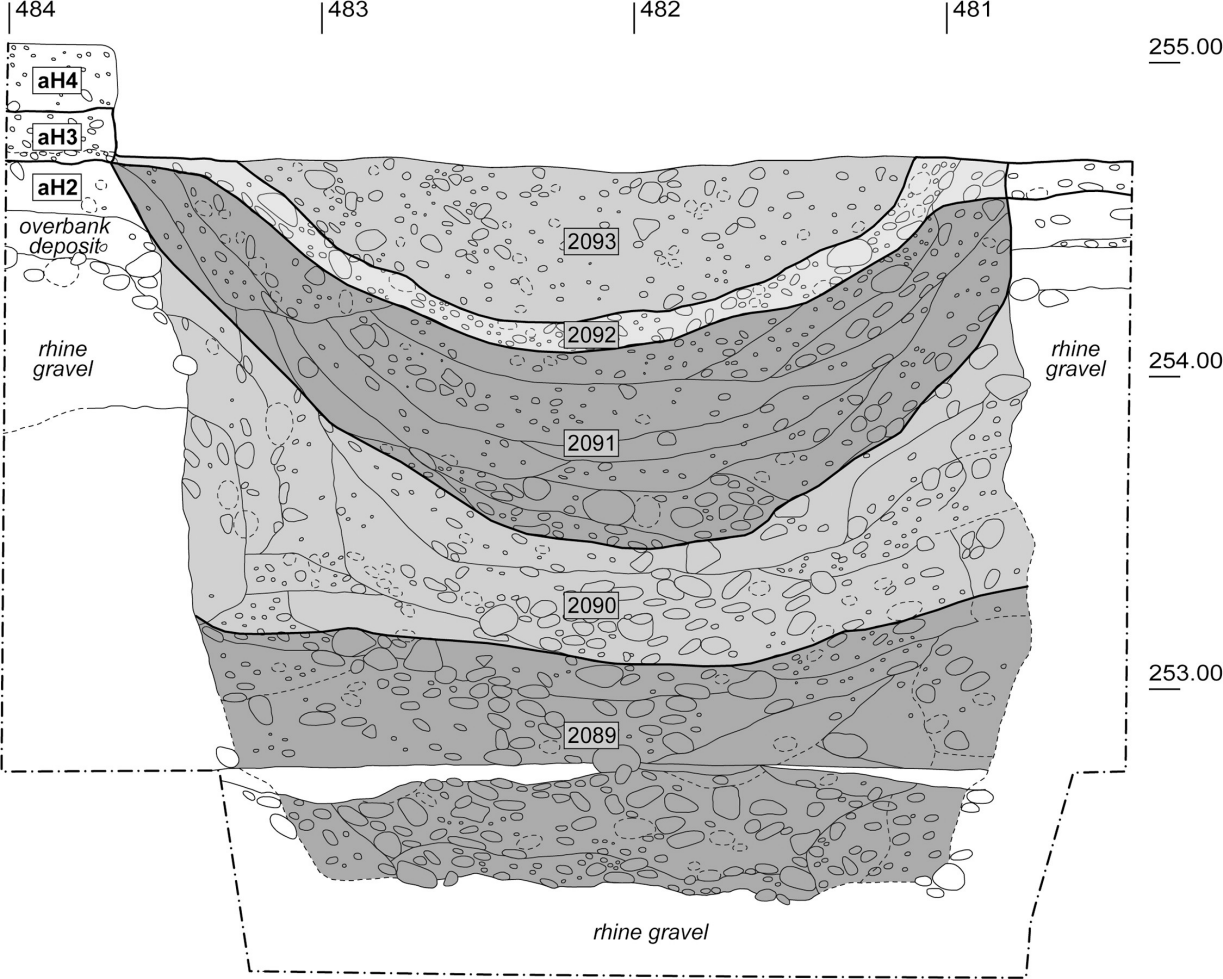
Pit 287. South section through pit 287 showing the different stratigraphic units (SU 2089–2093), which combined several layers each and were based on the results from the geoarchaeological and archaeological examinations.

Pit 287 was round, had a maximum diameter of c. 3 m and a depth of 2.2 m (Fig 4). Because its walls were vertical, we can assume that it contained some kind of stabilising internal structure, since the sandy Rhine gravel is very unstable and the pit walls would have collapsed within a short time otherwise. Its first fill after it had become redundant as a storage or cellar pit consisted of a sediment containing charcoal, large quantities of finds and a high proportion of gravel and debris (SU 2089). It then probably lay idle for a while, as indicated by a natural accumulation of amphibian remains (resulting from the animals falling into the pit and then being unable to climb back up its steep walls). The internal structure was also removed, which led to a partial collapse of the surrounding overbank deposit and Rhine gravel. A finely laminated sequence of ash layers was identified in the middle section of the pit fill (SU 2090). Such well-preserved, finely layered deposits are usually found in roofed areas where they are associated with successive accumulation by repeated trampling—in the case of ashes and charcoal often in the context of craftworking [30]. If the pit had been open and exposed to the elements, the resulting deposit, by contrast, would have been quite homogeneous. The pit was then completely filled with loam containing charcoal and numerous finds (SU 2091). A short while later, aH3 was deposited above it (SU 2092). The onset of compaction processes led to the layers subsiding, which in turn resulted in the formation of a shallow depression. This depression contained a coarsely bedded fill (SU 2093), which was interpreted as the result of the area being repeatedly levelled to counteract the subsidence [30].

### 2.3 Ditch 7

**Fig 5:**
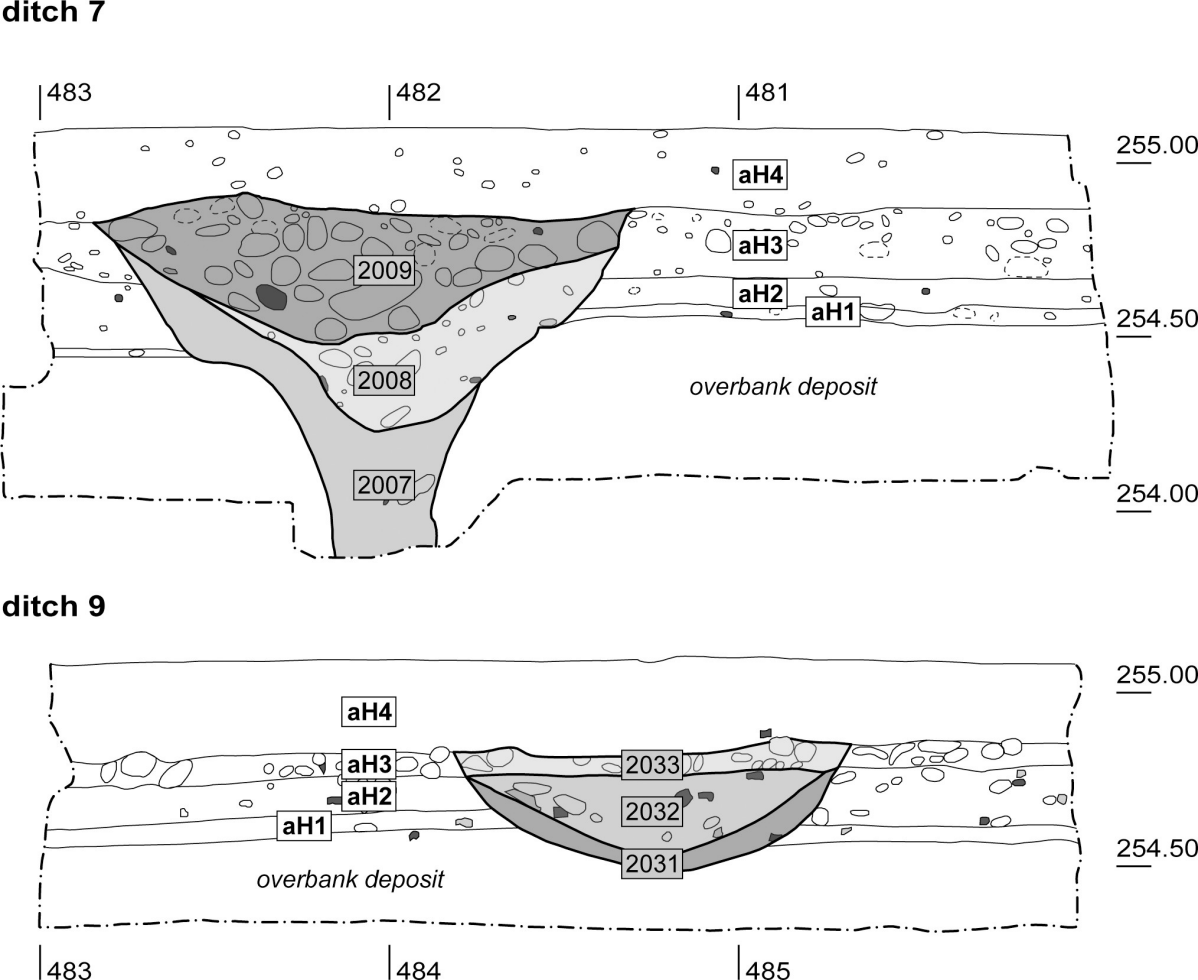
Transverse section through ditch 7 (top) and cross-section through ditch 9 (bottom). The ditch fills were divided into several stratigraphic units (SU 2007–2009 and SU 2031–2033). Grey objects: bones; dark grey objects: ceramic sherds.

Ditch 7 had a maximum width of 1.5 m (bottom width 0.3 m) and with its depth of up to 0.9 m reached down as far as the Rhine gravel in places. The multi-phased ditch was re-dug several times. In the bottom section, its walls were steep and clearly distinguishable from the base (Fig 5), suggesting that it had been lined. The upper flanks of the ditch, on the other hand, were clearly flatter and had over time probably eroded. The bottommost ditch fill (SU 2007) consisted of finely laminated loam, which had gradually accumulated and contained charcoal fragments as well as a small number of finds [30]. It was overlain by a fill consisting of a gravelly loam (SU 2008), which was heterogenous in nature without visible layering and must therefore have been introduced intentionally. The same can be assumed with regard to the following layer SU 2009 which consisted mainly of pebbles (Fig 5). It began beneath horizon aH3, but reached a considerably higher level, which is why it could not be ascertained whether or not SU 2009 and horizon aH3 were correlated.

### 2.4 Ditch 9

Ditch 9, which had at least two fills, was 0.4 m deep at most and between 0.8 and 1.0 m wide; it was trough-shaped in section (Fig 5). The lower fill (SU 2031), much like that of ditch 7, was interpreted as fine sediment that had been washed in naturally [30]. The overlying fill, which was heterogenous in nature and had thus probably been introduced intentionally, contained gravel and debris (SU 2032). This was followed by a layer of tightly packed pebbles, which was either another fill or perhaps part of aH3 (SU 2033).

## 3. Material and methods

The Archaeological Service of the Canton of Basel-Stadt (Switzerland) provided permits for the analysis of the archaeological material (ceramic sherds, animal bones, soil samples).

The reconstruction of taphonomic processes and of (regular) patterns of behaviour is a complex task that can only be tackled by using an integrative approach [28]. It should go beyond linear, interdisciplinary cooperation and be characterised by the fact that the research questions and the underlying theoretical concepts are jointly developed from the outset. The results obtained by the individual disciplines in this project were therefore not just brought together at the end of the work but discussed on an ongoing basis and at several meetings dedicated to formulating a synthesis and to continuously reviewing the hypotheses.

For the purposes of examining the taphonomic processes, various proxies were defined by each of the disciplines (pottery analysis, archaeozoology and geoarchaeology), which could be evaluated using the material available. The main proxies are listed in Tables 2, 3 and 4. A detailed and annotated list can be found in the supplementary information (S1 Table).

### 3.1 Pottery analyses (J. Wimmer)

Pottery is highly susceptible to taphonomic alteration because of its relative softness and because it progresses through its life cycles rather quickly and in substantial amounts. Although metal and glass objects are also interesting taphonomically thanks to their durability and recyclability, they were only present in relatively small numbers. We therefore decided to focus on the pottery fragments.

The pottery from the Late La Tène period can be divided into three types with distinctly different production methods, functions and origins [27]: (i) wheel-thrown fine ware produced in a kiln with a regulated atmosphere, (ii) handmade, pit-fired coarse ware and (iii) amphorae from the Mediterranean region. With regard to its function, we can state that fine ware, in contrast to coarse ware, was not usually used for cooking [9]. Fine ware was further subdivided by virtue of its firing method into oxidised and reduced wares. The former bore partially painted surfaces. For the purposes of this study, taphonomic data were available from 5,947 ceramic fragments weighing a total of 61.7 kg (69% fine ware, 28% coarse ware, 3% amphorae). They were attributed to 864 vessels. Taphonomic proxies were recorded for each fragment separately. Most proxies were subjective in nature and their comparability with other examinations is therefore limited. The following proxies were recorded: fragmentation (as a function of fragment weight), surface preservation, presence of painting on oxidised fine wares, matching fragments, traces of burning, spalling and sherd type (rim, bottom or wall sherd) (Table 2). A series of other proxies were not considered for various reasons, including rounded breaks, wear on vessel bases, phosphatic and carbonatic accretions and the high degree of fragmentation of the amphorae (S1 File).

### 3.2 Animal bone analyses (B. Stopp)

Taphonomic data were available from a total of 11,721 animal bones weighing 94.3 kg. Just over half of the bones were retrieved from the archaeological horizons (51.8%), approximately one third came from pit 287 (33%) and 15.3% were found in ditches 7 and 9. The taphonomic proxies recorded were traces of burning, gnawing marks, surface preservation, rounded edges, root damage and the average weight of the cattle bones (representing the degree of fragmentation) (S1 Table). Evaluation of the proxies examined was mainly based on the presence or absence of a proxy and on ordered categories (Table 3). Other taphonomic proxies were also recorded but were not included in the study for various reasons (too rare, not consistently assessed etc.). This included information on fatty aspects, digestion marks and postdepositional accretions and crusts.

### 3.3 Geoarchaeological analyses (D. Brönnimann)

A total of 50 block samples from 25 profile sections were micromorphologically examined by means of 89 thin sections. They covered 124 layers from three settlement pits (pit 287, 302 and 400), three ditches (ditches 4, 7 and 9) and 16 profile sections. In order to keep the database as large as possible, all micromorphologically examined features were included in the analysis of the taphonomic alterations.

The block samples from the sections were dried, impregnated with epoxy resin and polished sections created using a diamond saw. The 30 μm thick covered thin sections were prepared in Braunschweig (Germany), Caen (France) and Basel (Switzerland). The analysis was carried out using an optical microscope (Leica DM-RXP) with a polarisation filter and 16-630x magnification following the guidelines developed by George Stoops [37] and Mary-Agnès Courty and others [38]. The layers were analysed for their texture, fabric and composition. The components (bone fragments, ash etc.), structural phenomena (compaction, horizontal alignment etc.) and the postdepositional processes (bioturbation, precipitations etc.) were semi-quantitatively or qualitatively recorded using a 6-step scale. In addition, the bioerosion and collagen content of the bone fragments in the samples were assessed [29]. A total of 136 proxies were evaluated for each layer. This formed the basis for the reconstruction of the depositional and infilling processes (see chapter 2 of this paper). Eight proxies were chosen for the evaluation, all of which can be linked to various taphonomic processes (Tables 4 and S1).

## 4. Proxies and processes

In order to be able to compare the formation of the taphonomic features (proxies) examined by the different disciplines, each proxy was linked to one or more pre-defined processes (Table 5), which could have been involved in the formation of the proxy concerned. Only those processes that tend to leave visible traces on the objects were assessed. The focus in defining these processes (Table 5) was not on an object’s primary use but on what occurred after it had ceased to be used and before it ended up in its final depositional context.

The second step involved estimating (weighting) how much or how often each proxy recorded was impacted by each process (Tables 6 and S2). The weighting scale ranged from 1 (the proxy is only slightly/rarely impacted by the process) to 3 (the proxy is severely/often impacted by the process). Positive values indicate that a process supports / enables the formation of a proxy, whilst a negative value means that a process prevents a feature from developing (Table 6). Weighting and linkage were largely based on experience and estimates because experimental archaeological studies are rare and very few include any quantitative specifications (e.g. experiments on the redeposition and fragmentation of objects by trampling [39–43], the taphonomic changes to pottery caused by water and frost [44, 45] and on the alteration of sediments by trampling [46–49]. Moreover, the intensity of the taphonomic processes is closely linked to site-immanent factors such as the hardness or permeability of the substrate [41, 49] or the quality of the pottery [40] etc. Because of the lack of experimental data, the estimated weighting of the proxies was evaluated using statistical methods.

### 4.1 Confirmatory factor analysis (CFA)

**Fig 6:**
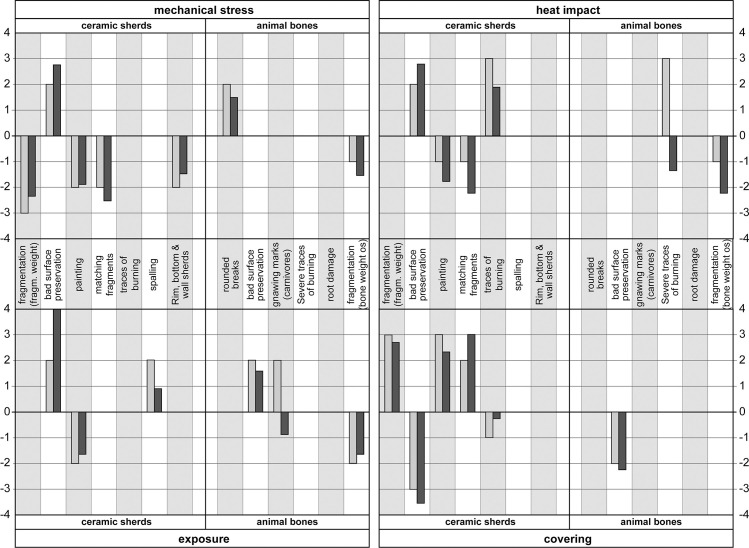
Confirmatory factor analysis. Comparing the weighting factors (proxy; grey) with the loadings of the confirmatory factor analysis (CFA; dark-grey) for the four processes with the most associated proxies (mechanical stress, heat impact, exposure, covering). To improve comparability, the loadings of the CFA were scaled to the sum of the proxy weighting factors (S2, S3, S4 and S5 Files).

One possible way to test the weighting postulated is to statistically analyse whether the proxies behave the same way in all stratigraphic units. This was done by carrying out a confirmatory factor analysis (CFA; *R-package “lavaan”*) [50]. For this, only those processes that could be linked to more than five proxies were considered (mechanical stress, heat impact, exposure, covering) (Fig 6) as there is limited information available on the other processes (primary use, water, redeposition, postdepositional processes). The test revealed that the weightings and the CFA loadings were congruent in most cases. Two exceptions were identified: the impact of heat on animal bones differed from that on pottery (process: heat impact) and gnawing traces on animal bones did not appear to be synchronous with the other exposure proxies. The former suggests that pottery and animal bones were subject to different secondary uses and were therefore not exposed to the same sources of heat. The latter probably indicates that the source of the gnawing traces–probably dogs–is a highly dynamic creature and carries a lot of intensity, but only acts it out on bones. In those cases, the opposite behaviours of the proxies can be explained by a different (heat) source, time period or place, so that the discrepancies do not contradict the weighting of the proxies.

Moreover, the values of the CFA loadings also often corresponded with the estimated weighting to within half a point (Fig 6). In two cases, the CFA led to the weighting being adjusted by a point (spalling in the case of exposure and heat impact in the case of rapid covering, S2 Table). Overall, however, the CFA confirmed the estimated weightings.

### 4.2 Statistical analysis

**Fig 7:**
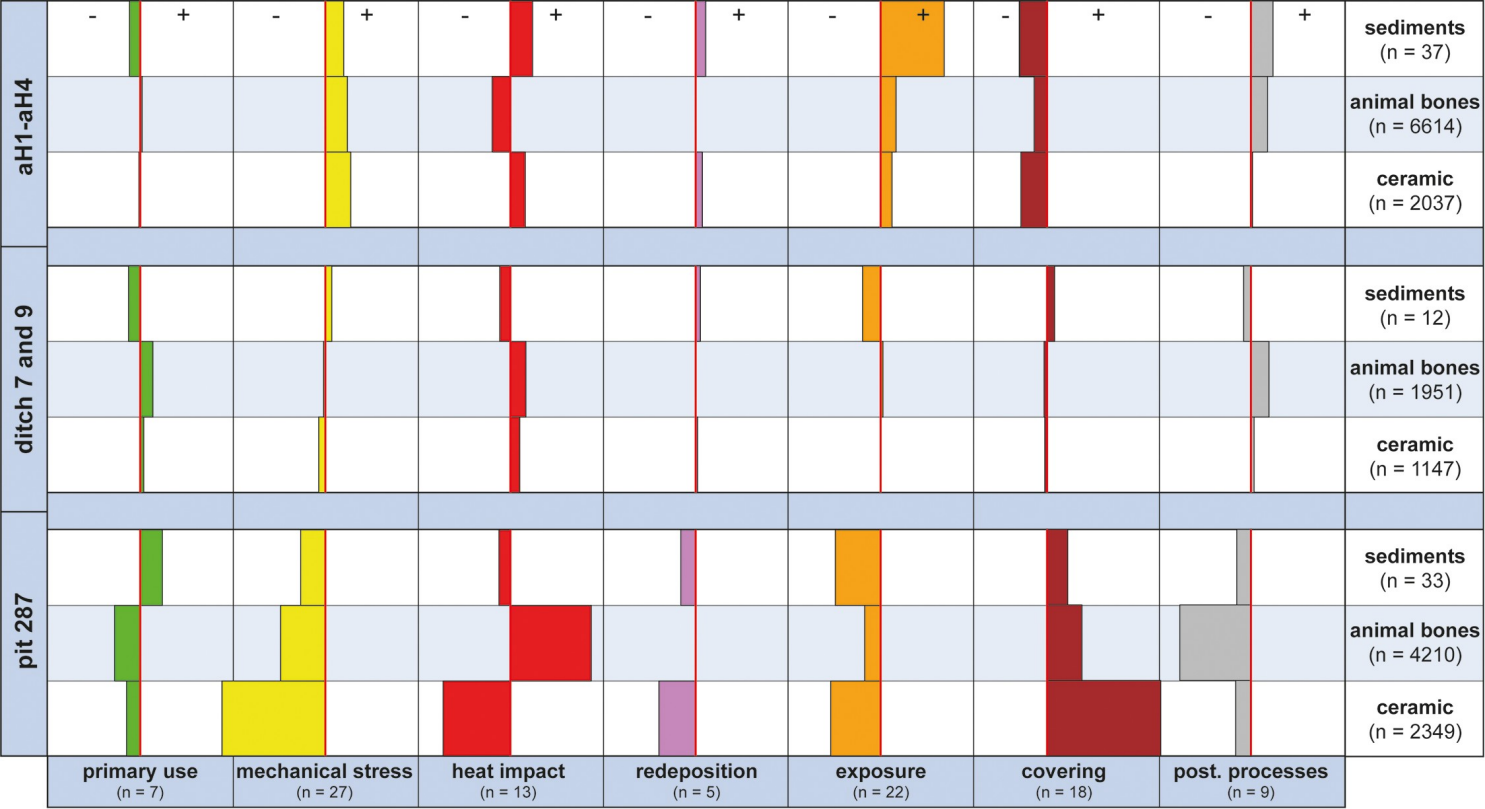
Comparison of the features examined. The seven taphonomic processes compared for pit 287, the two ditches and the four archaeological horizons, arranged by ceramic sherds, animal bones and sediments. Bars to the left (-) show slight/rare manifestation, whilst bars to the right (+) signify strong/frequent manifestation of the process. The axial red line marks the average manifestation. The sum of weighting marks for each process is given in brackets.

**Fig 8:**
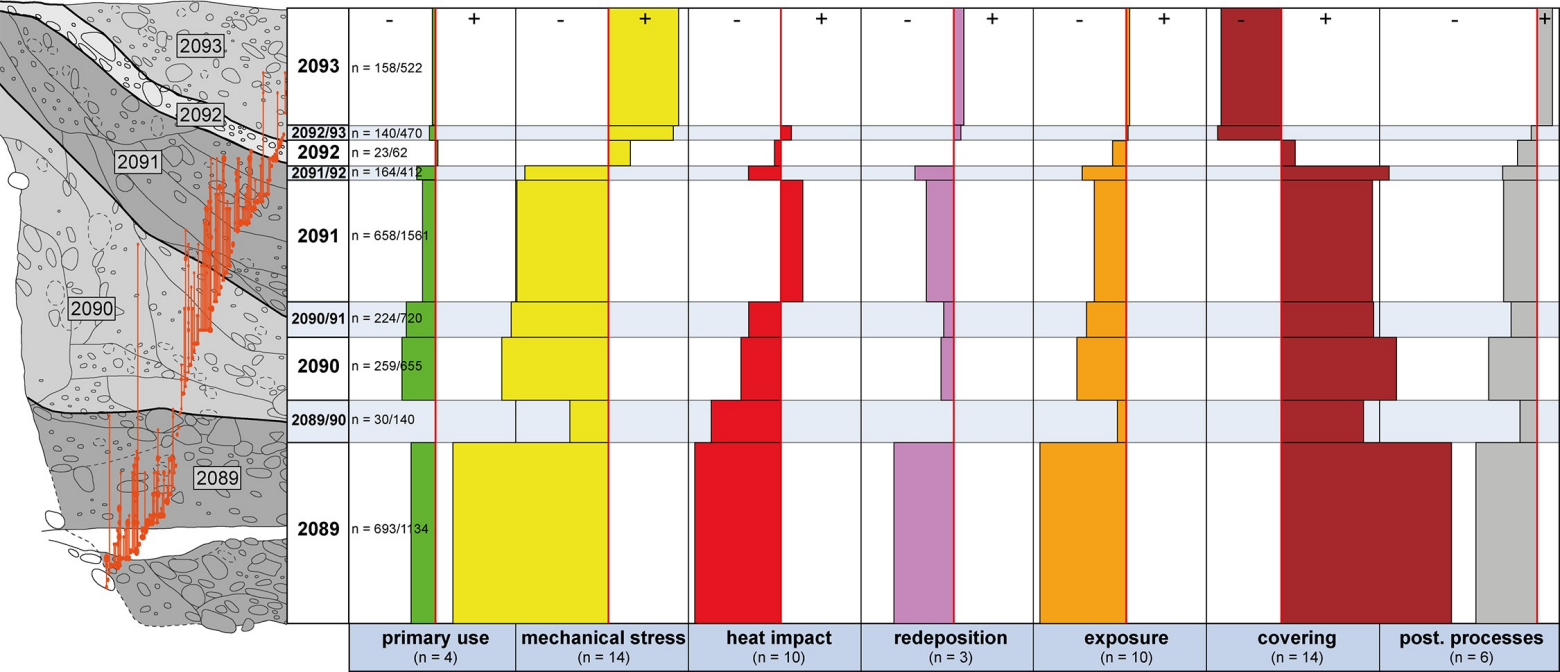
The impact of the taphonomic processes on the different fills (stratigraphic units) of pit 287 (pottery and animal bones combined). Bars to the left (-) show slight/rare manifestation, whilst bars to the right (+) signify strong/frequent manifestation of the process. The orange dots and lines in the image on the left denote joins between ceramic fragments.

**Fig 9:**
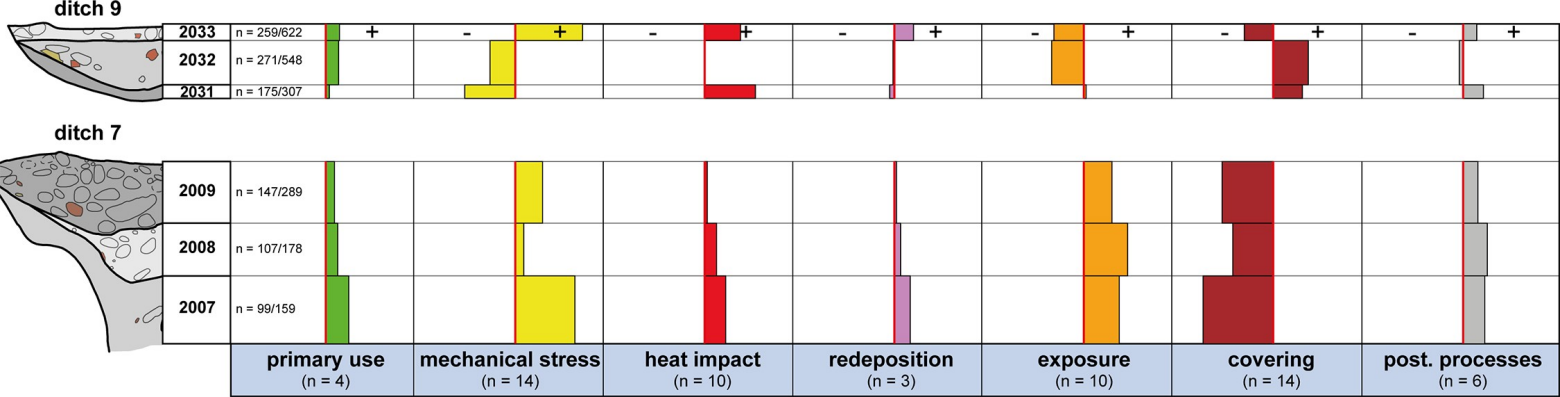
The taphonomic processes and their impact on the different infills of ditch 7 and 9 (pottery and animal bones combined). Bars to the left (-) show a slight/rare manifestation, whilst bars to the right (+) signify a strong/frequent manifestation of the process.

**Fig 10:**
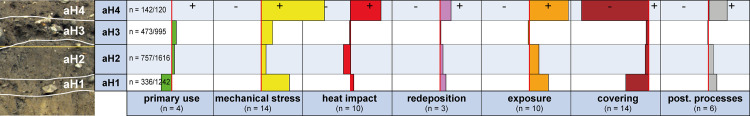
The taphonomic processes and their impact on the different archaeological horizons aH1-aH4 (pottery and animal bones combined). Bars to the left (-) show a slight/rare manifestation, whilst bars to the right (+) signify a strong/frequent manifestation of the process.

Within the stratigraphic units, the frequencies of attributes were calculated for categorical data and the averages for metric data. The former were transformed by integer scaling, which comprised the multiplication of the attribute frequencies by continuous integer factors. For example, in the case of “traces of burning” the frequency of the attribute "none" was multiplied by 0, "uncertain" by 1, "poor" by 2, "mean" by 3 and "intense" by 4. By summing up these multiplication products, a pseudometric mean could be calculated. This data transformation produced a (pseudo) metric value for each proxy. In order to maintain comparability, the data were standardised by proxy (z-transform). These values were then multiplied by the weighting outlined above, which produced scores for the individual processes and stratigraphic units (Figs 7–10). In this system, the sum of the weighting marks is a measure of the visibility of each process. We have refrained from scaling the scores yet again by the weighting marks because the traces left behind by some processes are less visible than those left by others. The process scores therefore mirror not just the intensity of the process itself but are also influenced by its visibility on archaeological sources. Because different numbers of proxies were recorded for each of the material categories (pottery, animal bones and sediments), they also have different weighting marks (pottery: 42, animal bones: 17, sediments: 40) and thus impact the process scores to varying degrees.

## 5. Results

### 5.1 Comparing the feature types

Comparing the proxies between the different features (large pit 287, ditch 7, ditch 9 and archaeological horizons aH1-aH4) revealed significant differences in the taphonomic alteration of the animal bones, ceramic sherds and sediments (Fig 7). Although there were some discrepancies, the different material categories react similarly in most processes (mechanical stress, redeposition, exposure, covering, postdepositional processes). However, the pottery and bone fragments behaved differently concerning their primary use and the influence of heat or fire. Another insight was that the artefacts and sediments in pit 287 exhibited the least taphonomic alteration (= best state of preservation), which was manifest in low scores in the case of mechanical stress, exposure, redeposition and postdepositional processes. The pottery and bone fragments and the sediments from archaeological horizons, on the other hand, provided the opposite picture, i.e. an intensive taphonomic alteration (= poor state of preservation), whilst the material from the two ditches, finally, bore average taphonomic alteration.

### 5.2 Pit 287

Because the geoarchaeological samples available from pit 287 were quite limited in number, only the results for the pottery and animal bone fragments were included in the study presented here. The same applies to ditches 7 and 9 (where only the bottommost, natural fills SU 2007 and SU 2031 were micromorphologically examined) and to the archaeological horizons.

As we had seen in other geoarchaeologically examined pits [30, 51, 52], the fills in pit 287 were quite heterogeneous. This fact was also reflected in the taphonomic alteration (Fig 8). Some of the fills bore significant differences, especially between the lower (SU 2089–2091) and the upper (SU 2092–2093) fills, which were largely congruent with the geoarchaeological data and the results obtained from the analysis of conjoining sherds (Fig 8). Taphonomic alteration was very slight in the lower section, particularly in respect of mechanical stress, redeposition, exposure and postdepositional processes, whilst the pottery and animal bones in the upper fills exhibited severe taphonomic alteration. This was consistent with the geoarchaeological results, which suggested that the lower section had been intentionally and rapidly filled, whilst the upper section was more likely to have been gradually filled with material by repeated processes of levelling. Natural sedimentation processes (washed-in sediments) can be excluded, since all characteristics are missing (e. g. fine-grained, laminated deposits with sizing of the (micro-) finds).

Another difference, though not quite as distinct, was visible between the bottommost (SU 2089) and the second-to-last (SU 2090–2091) stratigraphic unit. The taphonomic proxies in SU 2090–2091 were somewhat more distinct than in SU 2089. At the same time, it became obvious that whilst there were numerous conjoining sherds within SU 2089 and within SU 2090–2091, only a single join could be found between the two fills. These were separated by SU 2089/2090, which exhibited a higher degree of alteration by exposure and by mechanical stress and increased postdepositional processes. Moreover, a strikingly large number of amphibian bones were identified in this area. The middle stratigraphic unit in pit 287 (SU 2091) differed from the lower fills in that its material bore more intense signs of burning.

### 5.3 Ditch 7 and ditch 9

The examination of the taphonomic proxies of pottery and animal bone revealed significant differences between ditches 7 and 9 (Fig 9). The finds from ditch 7, for instance, exhibited signs of higher mechanical stress and exposure with lower values for covering than the artefacts from ditch 9. At the same time, some of the objects from ditch 9 bore traces of severe heat impact (burning). Finally, the proxies linked with primary use, redeposition and postdepositional processes were about average. Another difference between ditches 7 and 9 became manifest when comparing the ditch fills. Ditch 7 produced a rather homogenous picture with very few differences between the fills with regard to taphonomic alteration. The finds from the topmost fill (SU 2033) in ditch 9, on the other hand, bore traces of far more severe mechanical stress but compared to the bottommost fill (SU 2031) less alteration due to exposure (Fig 9).

### 5.4 Archaeological horizons (aH1-aH4)

Although the geoarchaeological data pointed to very different layer formation processes [30], the taphonomic alteration identified on the pottery and bone was quite homogenous for all horizons aH1-aH4 (Fig 10). The scores for mechanical stress and exposure were above average, whilst the score for covering came in below average (Fig 10). This trend became even more marked in the modern-period plough horizon aH4, which also showed a significant increase in the postdepositional processes. Moreover, the bottommost archaeological horizon aH1 scored slightly higher with regard to mechanical stress and exposure than aH2 and aH3.

Another aspect worth examining was whether there were any differences with regard to the taphonomic alteration of the pottery and animal bone fragments between the eastern and western sections of aH2. No clear pattern was detected, though individual differences were indeed identified (S6 File).

## 6. Discussion

### 6.1 Assessment of the different processes

Our examinations revealed that macroscopic analyses of ceramic sherds and animal bones mainly detected the effects of physical impact, whilst other processes such as primary use or redeposition were quite difficult to identify. In addition, micromorphological analyses can trace geochemical processes such as the preservation of collagen in bone.

From a methodological point of view, one could ask the question whether the connections we made between individual proxies and pre-defined taphonomic processes (Tables 5 and 6) were correct and coherent. An important clue to suggest that our connections were correct was the fact that the ceramic sherds, animal bones and sediments produced parallel values in respect of the different taphonomic processes (Fig 7). This was also confirmed by the confirmatory factor analysis (CFA) (Fig 6) (positive or negative values).

A large number of taphonomic features (proxies) were linked with the categories of “mechanical stress”, “exposure” and “redeposition processes”. These three processes largely ran in line with each other and were subsumed for the purposes of this study under the term “physical alteration”. This synchronicity makes perfect sense and can be seen as further evidence to support the coherence of our assessments. Moreover, it was obvious that this “physical alteration” usually ran counter to the process of “rapid covering”. It is worth noting, however, that this reciprocal picture was partially based on the fact that some proxies were associated with both the process of exposure and the process of rapid covering, albeit with reversed algebraic signs. The results also allowed us to conclude that “physical alteration” of bone, pottery and sediments occurs when exposed to everyday settlement activities. At the same time, objects which were (quickly) covered and thus withdrawn from everyday life exhibit distinctly weaker proxies. One exception to this rule was the impact of heat (fire), which only bore limited similarity with the “physical alteration” and was heterogenous even within individual archaeological features. We must therefore postulate a different treatment for the objects that had been impacted by fire.

### 6.2 Comparisons between and within the different archaeological features

Distinct differences were identified between pit 287, ditches 7 and 9 and the archaeological horizons aH1-aH4 with regard to taphonomic alteration. Based on earlier geoarchaeological and archaeozoological investigations carried on different pits, the hypothesis was formulated that archaeological objects found in pits are better preserved than those found outside of pits [30, 51, 53]. This assumption seems to have been confirmed by the taphonomical examinations carried out on pit 287, although it must be noted that the data is very limited.

#### Archaeological horizons aH1-aH4

The taphonomic alteration recorded on the finds from the archaeological horizons aH1-aH3 was quite severe but relatively homogenous (Fig 10). At first glance this came as a surprise, since the layers exhibited significant differences with regard to their characteristics and formation processes [30]. We can therefore assume that a large part of the taphonomic alteration is not just determined by how a layer is formed but also by what an object has undergone during its “everyday existence in the settlement”.

#### Pit 287

The strikingly good state of preservation observed in the objects from the lower sections of pit 287, in contrast to those from the archaeological horizons, was due to the fact that they were quickly deposited, covered and thus withdrawn from everyday use at the settlement. Such intentional and rapid pit fillings with fragmented coarse pottery, recorded in two other pits (pits 283 and 321) [28, 52], would have required large amounts of soil (sediments), ceramic sherds, bone fragments and other materials (slag etc.) being available at that particular time. We have therefore put forward the hypothesis that material stores existed where pottery sherds, animal bones and other waste material from “daily life” like iron slag, hearth plates and oven fragments were stored. Moreover, micromorphological examinations of pits in the settlement have shown that the associated sediment often contained charcoal and charred organic material, ashes, faeces (human, dog, pig) and decomposed daub [30]. Rather homogeneous mixing of all these materials was also observed in pit 287 and taken as an indication that these resources were not stored separately but intermixed without a discernible pattern. Therefore, a midden-like character can be postulated for these material stores. The good state of preservation of the objects from pit 287 suggests that these material stores were only rarely exposed to settlement activities or not at all and must therefore possibly have been kept in a sheltered location. The presence of gnawing marks on animal bones, however, indicates that these middens were at least temporarily accessible.

The almost complete lack of conjoining sherds and slight differences in the taphonomic alteration between the bottommost (SU 2089) and middle (SU 2090 and 2091) fills of pit 287 indicate that at least two different “material stores” were used to rapidly fill it in. This is further supported by the proposed short-term hiatus in the filling process between SU 2089 and SU 2090. Similar scenarios with hiatuses and changing sources of material were identified for pits 283 and 321, which have been studied as part of a separate interdisciplinary project [28].

Another phenomenon, which was also observed in pits 283 and 321, was seen in the top third of the pit fill (here SU 2092 and 2093): arched and sunken layers and strong taphonomic alteration of the pottery and animal bone fragments (Fig 8) [27, 9, 30, 32]. This was probably due to gradual processes of subsidence and repeated levelling, which means that the objects in the topmost fills lay exposed for certain periods of time and were thus subjected to settlement activities. This clearly shows that the pit fills at Basel-Gasfabrik should not be treated as homogenous units. The same applies to the pits themselves: although previous investigations revealed certain similarities in the filling processes, there were always differences, so that each pit must be viewed as a single “individual” with its own biography.

#### Ditches 7 and 9

The results from ditches 7 and 9 clearly show that ditches–much like pits–are by no means an homogenous type of feature. This was manifest not just in their shapes and depths (Fig 5), but also in the discrepancies in their taphonomic alteration, which differed significantly from that of pit 287. Both ditches contained the same fine-grained bottommost fill, which was mostly devoid of finds and consisted of washed-in surface material (SU 2007 and SU 2031), thus acting as a sediment trap. Microfinds such as drops of glass and bone splinters embedded in this layer can therefore provide clues with regard to the activities that took place in the immediate surroundings. The upper fills, on the other hand, were of a different nature and can be interpreted as intentional ditch fills (SU 2008, 2009 and SU 2032, 2033). The associated finds bore quite significant taphonomic alteration, which was heterogeneous both within and between the two ditches. The pottery and animal bone fragments from the fills thus did not (just) originate from the proposed material stores, since in that case one would expect them to be far better and more uniformly preserved. However, the taphonomic alteration of the objects from the two ditches was slightly less severe than that of the artefacts from the archaeological horizons. The material used to fill the ditches, besides numerous pebbles selected for their size, may have been a mixture of material from the proposed material stores (middens) and from “ordinary occupation layers”.

### 6.3 Differences between material categories

The taphonomic alteration of pottery and animal bone fragments due to “physical alteration” was approximately synchronous (Fig 7). One exception was the impact of heat (fire), which was quite heterogenous, particularly in the finds from pit 287. Because cooking or roasting meat hardly leaves any traces of burning on the bones and because fine ware is not generally exposed to fire in its primary use, traces of burning on animal bones and fine ceramic sherds can be traced back to a secondary use of these objects. In the case of the pottery, various reuses in a craftworking or domestic context (e.g. as an andiron, a support of some sort, a dipper, a hearth substructure or construction component etc.) come to mind [54–57]. Animal bones, on the other hand, may have been used as fuel: Whilst burnt bones were rarely found in the archaeozoological assemblage, micromorphological analyses revealed that the archaeological layers consistently contained severely burnt bone splinters, which suggests that animal bones were indeed used as fuel. The alteration by fire therefore showed that ceramic sherds and animal bones were reused in different ways. Despite this, there were numerous indications that animal bones and ceramic sherds were stored together rather than separately before they were deposited in the pits. One such clue was the consistently similar ratio between animal bones and ceramic fragments within the different fills of pit 287. Moreover, the fills of pit 287 (and also of pits 283 and 321) never contained separate layers of pottery or animal bones, but always a homogenous mixture of the two. Other material categories such as cattle mandibulae, which were used as sledge runners [58], however, appear to have been stored separately.

A specialised use was also observed in the third material group examined, i.e. in the naturally occurring overbank deposit loam and Rhine gravel (30), great amounts of which came to light anytime the ground was broken. The calcareous loam (C horizon overbank deposit) was used as daub, whilst the decalcified, clayey loam (Bt horizon overbank deposit) was utilised in the local production of pottery or in the construction of ovens or hearths [57]. Rhine gravel, on the other hand, was severely underrepresented and rarely occurred in the archaeological layers. One exception to this rule was layer aH3, which consisted of similar-sized large pebbles from the Rhine. This suggests that the pebbles were preselected for their size and then stored for a time.

### 6.4 The “invisible” objects

Although most pits yielded several thousand ceramic fragments and many conjoining sherds were found within each pit, it was hardly ever possible to reconstruct whole vessels. The same can be said for the animal bones: whilst the archaeological analyses showed that many of the pits yielded large minimum numbers of cattle, pig and sheep/goat individuals, each pit generally contained no more than 10% of an entire skeleton. This means that only a remarkably small proportion of the original vessel or individual animal skeleton ended up in any one pit. This raises the question of what became of the remainder. In contrast to organic material, which would have played an exceedingly important role in people’s everyday lives but which has not survived, neither the animal bones nor the pottery would have decayed completely.

In terms of the proposed material stores (middens) the obvious conclusion would be that the material was ultimately deposited in several different pits. However, the post-excavation work that has been carried out to date has rarely revealed conjoining sherds or animal bone fragments from different pits [9]. Distribution between various pits cannot therefore have been the only reason for the absence of material. Another possibility is that parts of vessels or carcasses remained out in the open and were incorporated in the archaeological horizons, given that the latter also yielded large quantities of both pottery and animal bone fragments. However, because join finding and refitting was hardly feasible in respect of the finds from the archaeological horizons due to the large quantities and poor state of artefact preservation, this theory is difficult to substantiate archaeologically. Yet another possibility is that some of the ceramic sherds or animal bone fragments were reused as raw material and almost completely destroyed in the process. Ceramic fragments, for instance, were used as grog in local pottery production [57], whilst bone was probably used as fuel. Finally, we must take into account the possibility that some of the pottery and animal bones (or other materials) were disposed of in a different manner that cannot be detected by archaeological means, for instance by dumping them in the Rhine or somewhere outside the settlement. For example, isotope investigations carried out on charred grain residues from Basel-Gasfabrik show that fields can be assumed to have been fertilised [59]. However, the amount of evidence found in the immediate surroundings of the settlement is not yet sufficient, so that the spreading of waste on fields to act as fertiliser, as known from medieval farming practices, seems unlikely [60].

### 6.5 Cultural biography of visible objects

The differences in the taphonomic alteration of the finds from pit 287 and the archaeological layers show that objects of the same type took different paths. After it had outlived its primary usefulness through being accidentally (or intentionally) shattered, at least part of the fine ware was deposited in presumed material stores (middens). The latter represent protected and “passive” zones, which means that the ceramic sherds and animal bones were accessible but did not go through any recognisable processes of transformation, unless they were retrieved for particular purposes and reused, for instance as fuel (bone), grog (pottery), for household chores or craftworking (which would have become manifest, for instance, in traces of secondary burning on ceramic sherds). At least some of these reused objects were subsequently deposited in the material stores for a second time and–along with material with hardly any taphonomic alteration–later used to (partially) fill a pit. We can assume that whole material stores, or perhaps just parts thereof, were “disposed of” in pit 287 and thus made inaccessible for further everyday use. Another proportion of the pottery and animal bone fragments, however, ended up in an “activated” zone (e.g. trampled surface) and was thus incorporated in the (turbulent) everyday life of the settlement. Whether the objects served a particular purpose (e.g. to reinforce a surface) or whether they “participated” in everyday life at the settlement, can no longer be ascertained. All we can say is that they experienced severe taphonomic alteration, which we subsume under the terms “physical stress” and “exposure”.

## 7. Conclusion

Our results showed that the biggest differences with regard to the taphonomic alteration of ceramic sherds, animal bones and sediments existed between the different archaeological features (pit 287, ditches 7 and 9 and archaeological horizons aH1-aH4). This was confirmed by the geoarchaeological results, which indicated that completely different processes had led to the formation of pit and ditch fills and archaeological horizons. The same can be said for the different fills of pit 287: the bottommost, rapidly introduced layers bore clearly less severe taphonomic alteration than the upper strata, which had been formed by repeated levelling. Moreover, the results from ditches 7 and 9 showed that ditches must be seen as heterogenous stratigraphic units from the point of view of taphonomic alteration. Moreover, we can also say that the ceramic sherds and animal bones deposited in archaeological horizons aH1-aH4 bore fairly homogenous and quite severe taphonomic alteration, although aH1, aH2 and aH3 were clearly very different both in respect of their properties and of their layer formation processes. Finally, it must be taken into account that every pit, ditch and archaeological layer has its own history and must therefore be assessed and investigated separately and seen as an independent "individual".

The synchronicity of the proxies that were associated with “physical stress” (mechanical stress, exposure, redeposition) suggests that a large part of the macroscopically visible taphonomical features of the pottery and animal bone fragments can be attributed to the fact that these objects were “integrated” into everyday life. This is only possible if objects lie on or near the surface (“passive” participation) or if they are explicitly reused (“activated” participation in everyday life). Both scenarios result in accidental or targeted manipulation of the objects. Alteration through fire (heat impact) is a different case in that it varies in severity within the individual features (particularly in the ditches) and between the ceramic sherds and animal bone fragments, thus probably indicating a secondary use of both pottery and animal bones.

The results from our taphonomic examinations suggest that pottery sherds, animal bone fragments and probably also sediments and other objects were kept in “material stores” (middens; primary or secondary refuse) and later deposited in pits (secondary refuse). Evidence to suggest that the material within pit 287 originated from different sources was seen as proof of the existence of several smaller material stores (middens). Apparently only a fraction of the vessel and of the animal skeleton ended up in the pits. The high minimum numbers of individuals both with regard to the pottery and the animal carcasses within pit 287 suggest that “dissecting” and “distributing” of vessels and skeleton individuals was not the exception but, rather, the rule, though neither the time nor place are known at which this probably multistage process took place. Accordingly, the individual sherds and bone fragments took different paths once the ceramic vessel had been broken or the animal had been slaughtered. Some were kept in material stores (middens) and then deposited in pits (secondary waste), some were integrated into everyday life at the settlement and severely altered (object recycling), stored as raw material and reused, or else completely transformed (grog) or destroyed (fuel) (material recycling) (Fig 1). This multi-branched path appears to have been a regular pattern and points to a clearly defined treatment of “waste” on the one hand and a complex and intricate cultural biography of things on the other.

The existence of (small) material stores (middens) as attested to by our study has far-reaching consequences for the interpretation of archaeological finds and raises new questions. This temporary storage of material represents yet another “passive phase” of unknown chronological and spatial depth. The good state of preservation of the finds from pit 287 (and pits 283 and 321), however, as well as the fact that the fills (in pit 287 in particular) consisted of several rather quickly introduced layers, suggests that the material stores were quite short-lived features. Another unknown feature is the spatial dimension–in other words the catchment area of each material store. In the case of smaller stores, the catchment area would probably have been quite limited, which would mean that the individual fills could be interpreted as chronologically and spatially rather narrowly defined assemblages. This hypothesis, however, requires further study. This subject notwithstanding, we can state that from a chronological point of view pit 287, ditches 7 and 9 and archaeological horizons aH1-aH4 of the settlement at Basel-Gasfabrik do not represent a closed context. In order to interpret the individual finds assemblages, it is therefore necessary to put the archaeological features and finds in a bigger context. One of the aspects that form part of this context, are the waste disposal practices, which had a considerable impact on the distribution and preservation of the finds and thus on the informative value of archaeological finds assemblages. It is possible, at least in part, to reconstruct the cultural biography of things by means of taphonomic analysis and collaboration between various disciplines. The results have shown that different material categories had different cultural biographies. What is true for a fragment of a fine-ware bottle does not necessarily apply to an animal bone, even if both objects originate from the same feature. It is therefore essential to evaluate not just each stratigraphic unit but also each material category as a separate entity. This will allow us to trace patterns, which is eminently important not just for the interpretation of finds assemblages but also for the perception of the out of the ordinary (e.g. of deposits).

In archaeology, the location and function of an artefact (or fragment) is often used to reconstruct (settlement) activities. However, the place of discovery of an object does not necessarily (and in fact it rarely does) correspond to its place of use, and the function of an object or fragment can change. Therefore, the evaluation of taphonomic processes can provide information about the (regular) treatment of object groups and materials and thus about site formation processes.

### Outlook

Our examinations have provided a novel view of the cultural biography of things based on the Late La Tène settlement Basel-Gasfabrik, which also raises many new questions. If we are to gain a better understanding of the archaeological find assemblages, it is essential to find out more about the chronological and spatial depth of the proposed material stores. It will also be interesting to include other material categories (amphora sherds, glass objects, non-ferrous metal objects, droppings, faeces etc.) in future studies.

Analysing taphonomic processes is time-consuming. However, our study has shown that not all proxies have the same significance. We consider the following proxies to be highly relevant: fragmentation, surface preservation, traces of burning (pottery, bones), gnawing marks (bones), fine layering or homogenisation (sediments), degree of fragmentation of charcoal and coprolites (sediments) and histotaphonomy (collagen content, fungal attack). It should be noted that the significance of proxies may depend on the setting of a site.

Furthermore, balancing effort and return is often crucial for archaeological investigations. In our experience, the fragmentation (e.g. based on weight) and traces of burning on ceramic sherds and animal bones are relatively easy to evaluate and have a high informative value. Both proxies provide readily comparable data and important information on physical and thermal alteration. Moreover, our study has shown that geoarchaeological and histotaphonomic results are difficult to compare with results from other disciplines due to differences in data quality and quantity. However, both methods provide essential information on sediment formation processes, which is crucial for the interpretation of (bio-) archaeological data.

In order to test the hypotheses presented in this paper, a greater amount of taphonomical data from different archaeological structures is required. It is essential to include the knowledge gained regarding the significance of proxies to develop a data base with as little effort as possible. We would suggest that the same procedure is followed for other projects: the first step should be to compile an extensive record of various proxies on the basis of a limited number of objects. The second step involves identifying the most meaningful proxies so that further investigations can be carried out using a simplified procedure.

## Supporting information

S1 Table Description of the analyzed proxies. (XLSX)

S2 Table CFA scores. (XLSX)

S1 File Explanation of the proxies for ceramics. (DOCX)

S2 File CFA code for the proxy “covering”. (TXT)

S3 File CFA code for the proxy “exposure”. (TXT)

S4 File CFA code for the proxy “heat impact”. (TXT)

S5 File CFA code for the proxy “mechanical stress”. (TXT)

S6 File Results archaeological horizon aH2 comparing the western and eastern area. (PDF)
