# Supplementary material for: One man’s trash is another man’s treasure. Interdisciplinary examination of taphonomic aspects of ceramic sherds, animal bones and sediments from the La Tène period settlement at Basel-Gasfabrik
Source: PLoS One. 2020 Jul 27;15(7):e0236272. doi: 10.1371/journal.pone.0236272 (PMC7384648; doi:10.1371/journal.pone.0236272)
Supplement: S1 File — (DOCX) [file pone.0236272.s003.docx]

**Detailed description of the taphonomic proxies for ceramics**

| **Proxy** | **Description** | | | |
| --- | --- | --- | --- | --- |
| **fragmentation of fine ceramics** (numeric) | Fragmentation was determined based on the weights of the fine ceramic wall sherds. Coarse ware as well as rim and bottom fragments were not taken into account because they are thicker-walled, meaning heavier at the same size, and more resistant to breaking. The weights of individual fragments were logarithmised in order to convert their skewed distribution into a normal distribution (see fig. #1). This transformation allows the calculation of mean and standard deviation. The determination of this proxy is objective.  In addition to their weight, the size of fragments can also be used to assess fragmentation, e.g. by measuring length or surface (Nielsen 1991; Furger-Gunti 1979). If one assumes that density and wall thickness remain more or less constant within the settlement, size and weight are linked to each other via volume and are likely to represent fragmentation on a comparable level. When collecting data, weight has the advantage that it can be recorded quickly, exactly and reproducibly.  In some cases, fragmentation may proceed postdepositionally as a result of sediment pressure, so that objects that entered the ground as whole vessels or as larger parts also suffer a certain fragmentation. For this, broken finds from depositions or broken grave goods may serve as an example (Niederhäuser et al., in prep.). | | | |
| **surface preservation of fine ceramics** (classes) | Preservation of fine ceramic surfaces on the outside of vessels, which were polished with a solid, smooth object during production, can be divided into five categories. The determination of this proxy is subjective.  The inner surface was not included, since this surface has not been polished and the data therefore cannot be compared unconditionally. Furthermore, these two surfaces do not show significant differences in preservation. Coarse ware was not taken into account because of differing surface treatment during production. | | | |
|  | luster | | intact, glossy surface | 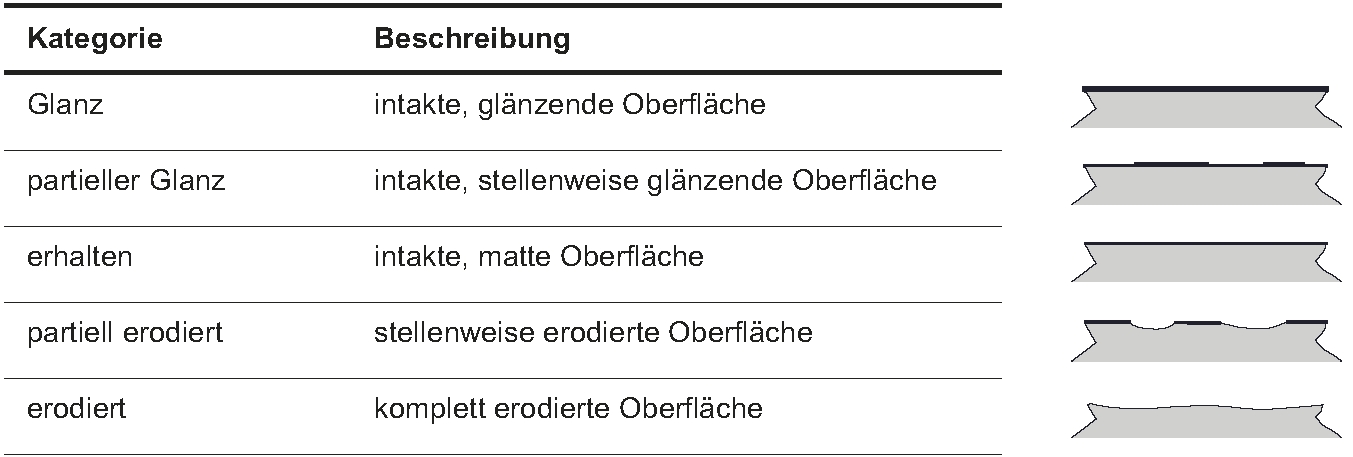 |
|  | partial luster | | intact, partially glossy surface |  |
|  | intact | | intact, but dull surface |  |
|  | partially eroded | | partially eroded surface |  |
|  | eroded | | completely eroded surface |  |
| **painting on oxidizing fired fine ceramics** (yes/no) | The presence or absence of painting on oxidizing fired fine ceramics was surveyed. Based on well-preserved vessels, it can be estimated that up to 80% of the surface of oxidizing fired vessels were painted. A lower frequency of painting on sherds therefore has to be a result of taphonomic transformation. The determination of this proxy is objective. | | | |
| **matching fragments of fine ceramics and coarse ware** (numeric) | To record matching of fragments, the number of conjoining sherds has been counted as well as the number of fragments most likely belonging to the same vessel. The frequency of these two proxies is essentially depending on how long one will search for matching fragments during data acquisition. It is therefore an objective, but not in any case comparable proxy. | | | |
| **traces of burning on fine ceramics** (classes) | The evaluation of heat impact on fine ceramics was carried out using five categories. Since fine ceramics are not cookware, it can be assumed that contact with fire usually took place after the primary use of the vessel. The changing of colours caused by the impact of heat (fig. #2) has not the same visibility, depending on whether reducing or oxidizing firing was carried out originally. The determination of this proxy is subjective. The impact of heat on coarse ware was not included, as it is not possible to distinguish its formation during the firing in a fire pit, during the use as cookware or after fragmentation. The latter can only be assessed based on heat traces at broken edges. | | | |
|  | none | no visible traces | | |
|  | uncertain | suspected | | |
|  | poor | only superficial changing of colours | | |
|  | mean | changing of colours inside the sherd | | |
|  | intense | deformation, slagging and/or swelling | | |
| **spalling on fine ceramics**  (yes/no) | The presence of spalling on fine ceramics, which orientate on the pores in the sherd and run parallel to its surface (Skibo et al., 1989), was recorded. Similar spalling can be observed nowadays on ceramics exposed to the weather (e. g. flowerpots). However, spalling can also occur when storing salty goods (Bonaventure, 2011). A distinction between these two causes is not always possible (fig. 3). The determination of this proxy is subjective. | | | |
| **frequency of rim and bottom sherds, coarse ware and fine ceramics**  (classes) | For each fragment, it was determined whether it is a rim, bottom or wall sherd. With increasing fragmentation, the probability increases that a wall sherd breaks off from a rim or bottom sherd. Depending on the structure and preferred breaking directions within the vessel, this leads to a higher percentage of wall fragments. The determination of this proxy is objective. | | | |

**Fig. #1:** Skewed distribution of sherd weights (left) and logarithmic transformation into a normal distribution (right), shown by a diagonal line in the QQ-plot (bottom).


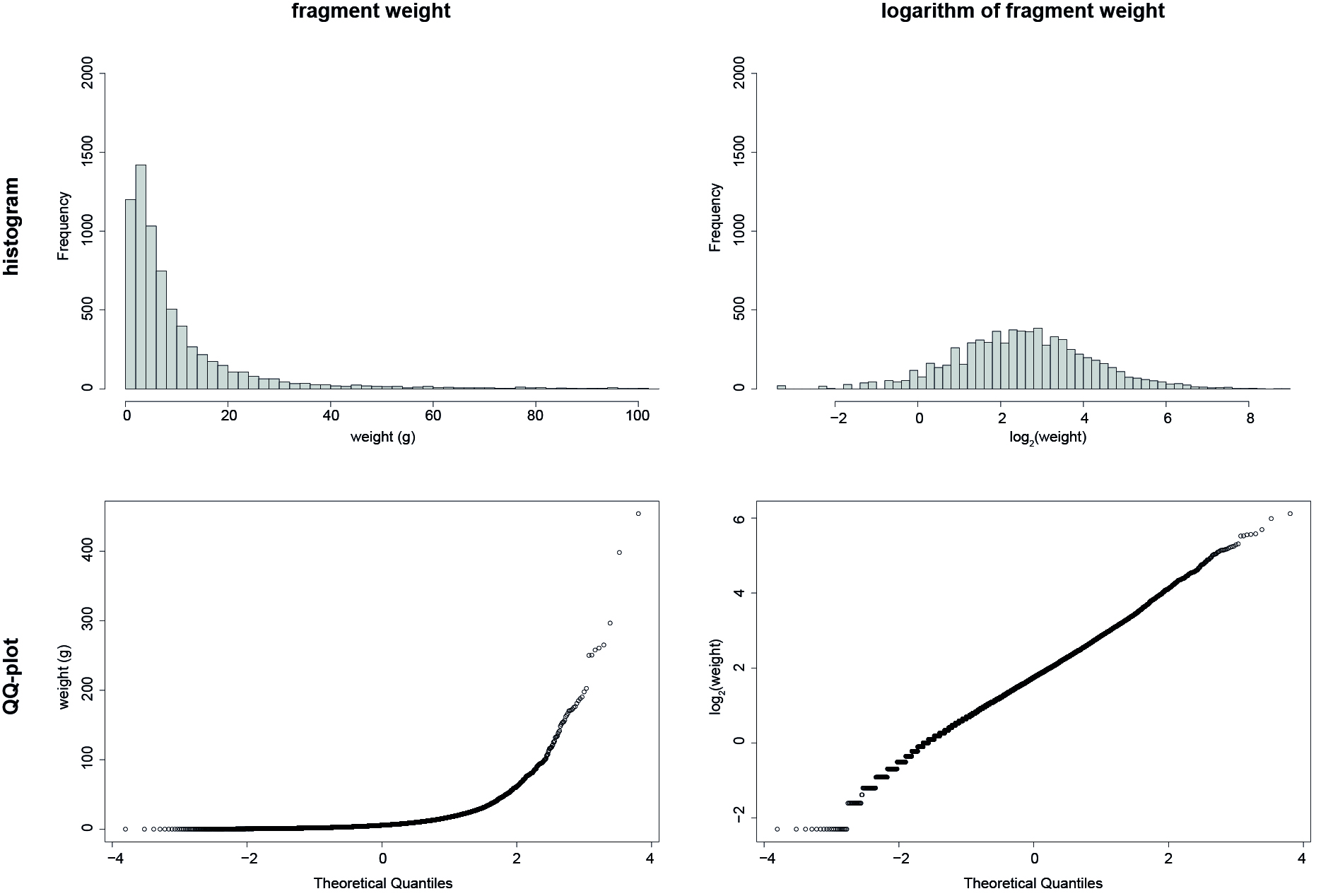


**Fig. #2:** Changing of colours caused by heat impact on different sherds belonging to the same vessel. Right: original colour, left: changed colour (blackening).


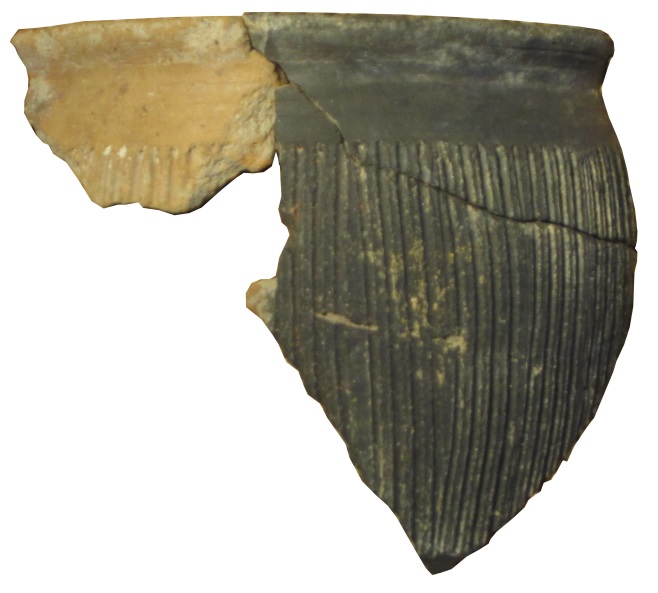


**Fig. #3:** Spalling on fine ceramics. A: spalling on the broken edges of a fragment (arrows), probably caused by weather exposure after fragmentation, B: spalling on the inner surface of a vessel that was perhaps used to store salty liquids.


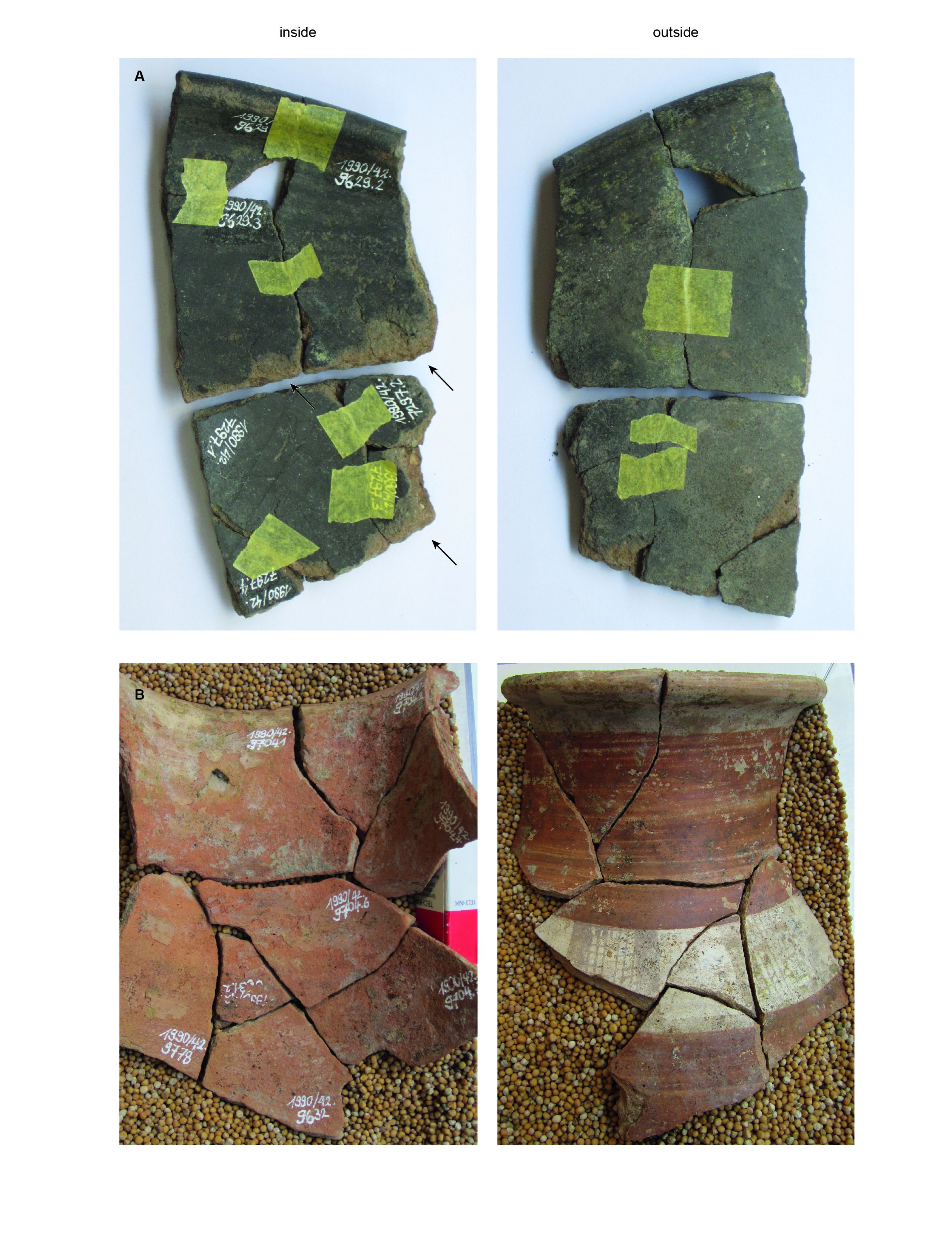


**References**

Bonaventure, B., 2011. Céramiques et société chez les Leuques et les Médiomatriques (IIe – Ier siècles avant J.-C.). Protohistoire européenne 13, 2011.

Furger-Gunti, A., 1979. Die Ausgrabungen im Basler Münster I: Die spätkeltische und augusteische Zeit (1. Jahrhundert v. Chr.). Basler Beiträge zur Ur- und Frühgeschichte 6. Habegger, Derendingen, Solothurn.

Niederhäuser, A.; Rissanen, H., Wimmer, J., in prep. Auf den Kopf gestellt. Eine aussergewöhnliche Deponierung in der jüngerlatènezeitlichen Zentralsiedlung Basel-Gasfabrik. Beiträge zur internationalen Tagung der AG Eisenzeit in Halle 2018.

Nielsen, A.E., 1991. Trampling the archaeological record: An experimental study. American Antiquity 56 (3), 483–503.

Skibo, J.M., Schiffer, M.B., Reid, K.C., 1989. Organic-tempered pottery: An experimental study. American Antiquity 54 (1), 122–146.
