# Supplementary material for: One man’s trash is another man’s treasure. Interdisciplinary examination of taphonomic aspects of ceramic sherds, animal bones and sediments from the La Tène period settlement at Basel-Gasfabrik
Source: PLoS One. 2020 Jul 27;15(7):e0236272. doi: 10.1371/journal.pone.0236272 (PMC7384648; doi:10.1371/journal.pone.0236272)

supplementary information 5 - H2 west/east

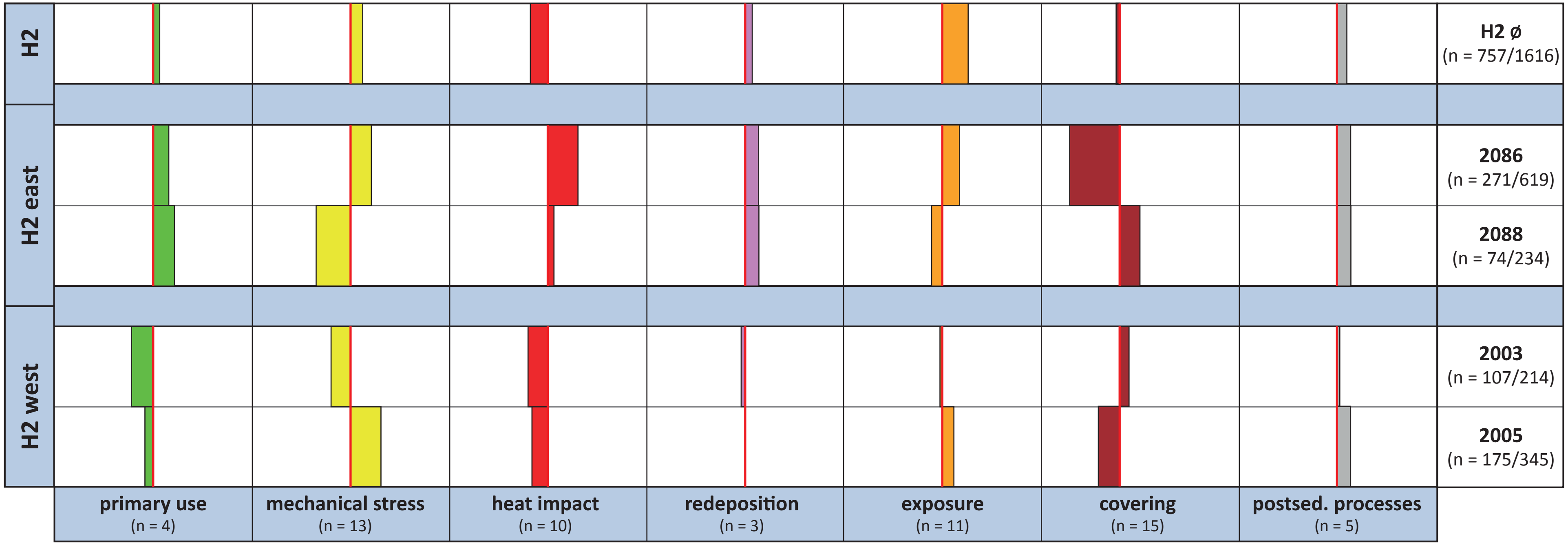

Supplement: S6 File — (PDF) [file pone.0236272.s008.pdf]
